# The morphological mapping of lateral compression type 1 pelvic fracture and pelvic ring stability classification: a finite element analysis

**DOI:** 10.1186/s13018-021-02818-3

**Published:** 2021-11-17

**Authors:** Bin-Fei Zhang, Jun Wang, Yu-Min Zhang, Hui-Guang Cheng, Qian-Yue Cheng, Wen-Wen Cao

**Affiliations:** Department of Joint Surgery, Honghui Hospital, Xi’an Jiaotong University, No. 555 Youyi East Road, Beilin District, Xi’an, Shaanxi Province 710054 The People’s Republic of China

**Keywords:** Morphological mapping, Lateral compression type 1, Pelvic fracture, Stability, Classification, Finite element analysis

## Abstract

**Purpose:**

This finite element analysis assessed lateral compression (LC-1) fracture stability using machine learning for morphological mapping and classification of pelvic ring stability.

**Methods:**

Computed tomography (CT) files of LC-1 pelvic fractures were collected. After morphological mapping and producing matrix data, we used *K*-means clustering in unsupervised machine learning to classify the fractures. Based on these subtypes, we manually added fracture lines in ANSYS software. Finally, we performed a finite element analysis of a normal pelvis and eight fracture subtypes based on von Mises stress and total deformation changes.

**Results:**

A total of 218 consecutive cases were analyzed. According to the three main factors—zone of sacral injury and completion, pubic ramus injury side, and the sagittal rotation of the injured hemipelvis—the LC-1 injuries were classified into eight subtypes (I–VIII). No significant differences in stress or deformation were observed between unilateral and bilateral public ramus fractures. Subtypes VI and VIII showed the maximum stress while subtypes V–VIII showed the maximum deformation in the total pelvis and sacrum. The subtypes did not differ in superior public ramus deformation.

**Conclusions:**

Complete fracture of sacrum zones 2/3 may be a feature of unstable LC-1 fractures. Surgeons should give surgical strategies for subtypes V–VIII.

## Introduction

Lateral compression type-1 (LC-1) pelvic fractures account for approximately 50–60% [1–3] of all pelvic injuries. However, the understanding of LC-1 is controversial, especially in terms of pelvic ring stability and treatment strategies [4]. Owing to differences in factors that affect the impact site and speed, the manifestations of LC-1 fractures are diverse and the pelvic ring stability varies widely. Investigators previously believed that LC-1 fractures were unstable and recommended surgery [5]. Subsequently, some investigators suggested that partial posterior ring ligaments and the pelvic floor structure in LC-1 remained intact and that these fractures were stable [6]. Thus, conservative treatment of LC-1 fractures was recommended [7]. Recent studies have shown that LC-1 fractures are highly variable pelvic injuries, with some clustering as stable and others as unstable [8].

However, how to accurately determine the stability of the pelvic ring in LC-1 pelvic fractures remains uncertain. Parry et al. have found weightbearing as tolerated could not identify all cases of occult instability, and resulted in an increased time to surgery for patients needing operative treatment [9]. Additionally, it is challenging to determine stability of the pelvic ring using static X-rays, computed tomography (CT), and magnetic resonance imaging (MRI). Ma et al. found the true instantaneous deformation of the pelvis was 2.2 times to the displacement at admission [10]. Gary et al. reported the use of MRI to scan anterior and posterior pelvic ligament injuries to predict pelvic stability [11]. However, this method is limited to an indirect strategy. Because of the lack of understanding of the LC-1 stability mechanism, orthopedic surgeons cannot evaluate pelvis stability from static images or formulate the optimal treatment strategies for these patients.

Due to these limitations, the study of dynamic biomechanical mechanisms has become the best way to determine the stability of the LC-1 pelvic ring. In the past 5 years, our team has used ultrasound to detect mobility of the superior pubic ramus under pelvic compression and separation tests to dynamically determine the stability of the LC-1 pelvic ring [12–15]. We have developed a standard operating procedure for testing [12, 13], quantified the criterion for stability [13], evaluated its accuracy [13], and followed up the long-term results [15]. However, there remain several limitations to the current judging system. The main limitation is the use of ultrasound to observe anterior ring mobility and indirectly assess pelvic ring stability, which fails to consider the fracture shape and degree of sacral damage. Therefore, the sensitivity and specificity of ultrasound diagnosis are low (66.67% and 76.92%, respectively) [13].

To overcome the current predicament of biomechanical study, we designed this finite element analysis based on the use of machine learning to draw the morphological mapping and classify subgroups of pelvic ring stability to assess the stability of LC-1 fractures.

## Materials and methods

### Intelligent classification of machine learning

We collected computed tomography (CT) files of LC-1 pelvic fractures in our hospital from 2015 to 2020. The section thickness was 1.5 mm. The images were imported into the computer system and the following steps were performed:

1. If the injured side of the pelvis was contralateral, we used the mirror function to show the fracture line on the identified side.
2. We drew the morphological mapping.
3. After completing the morphological mapping, we obtained the matrix data using K-means clustering analysis by applying an unsupervised machine learning method.
4. Based on the sacral fracture morphology, we classified LC-1 pelvic fractures.

### Finite element analysis

First, we screened DICOM data of regular pelvic patients from the CT database. Then, we used HyperMesh software (version 12.0; Altair Engineering, Michigan, United States) for pre-processing before performing the finite element analysis. Next, we imported the obtained geometries into ANSYS 19.2 (ANSYS, Inc., Canonsburg, PA, USA). Then, according to the subtypes from the machine learning classification, we manually operated models of fracture subtypes by adding fracture lines in ANSYS. Since this study focused on bones, we ignored the complex modeling of the ligaments. Therefore, all the contacts between the components were considered entirely bound to keep the pelvis assembled.

Before the finite element analysis, a patch-independent algorithm and quadratic tetrahedral elements were used for meshing. We set the Young's modulus E and Poisson's ratio ν of each component according to the parameters described previously [16]. The Young's modulus E and Poisson's ratio ν were 7000 Mpa and 0.3 for bones [17], 5 Mpa and 0.495 for the pubic symphysis [18], and 350 Mpa and 0.495 for the sacroiliac joints [19], respectively.

The conditions of the finite element analysis were as follows: the bilateral ischial tuberosity was fixed and a static force of 500 N was applied in the vertical direction. We performed finite element analyses of the normal pelvis and eight fracture subtypes, focusing on von Mises stress and total deformation changes. We then exported the von Mises stress and total deformation diagrams and assessed the reasonableness of the calculations, revising the results as necessary.

## Results

### Intelligent classification of machine learning

**Fig. 1:**
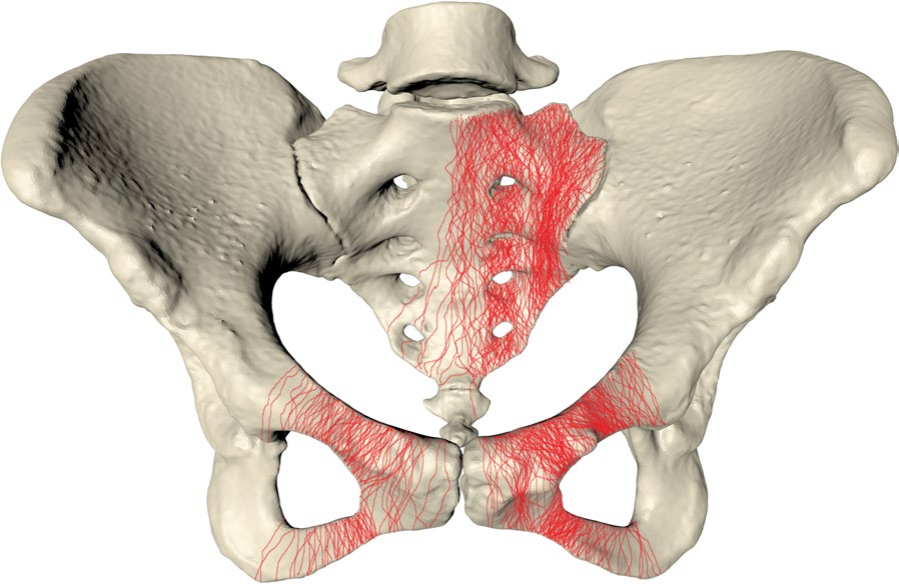
Morphological mapping of the fracture lines in LC-1 pelvic fracture. The lines are in the sacrum, ipsilateral and contralateral pubic ramus

This study collected 218 consecutive cases and their DICOM images from 2015 to 2020. The average patient age was 46.56 years, and there were 165 men and 53 women. We imported the CT data of LC-1 pelvic fractures into a computer system and drew morphological maps of the fracture line, as shown in Fig. 1.

**Fig. 2:**
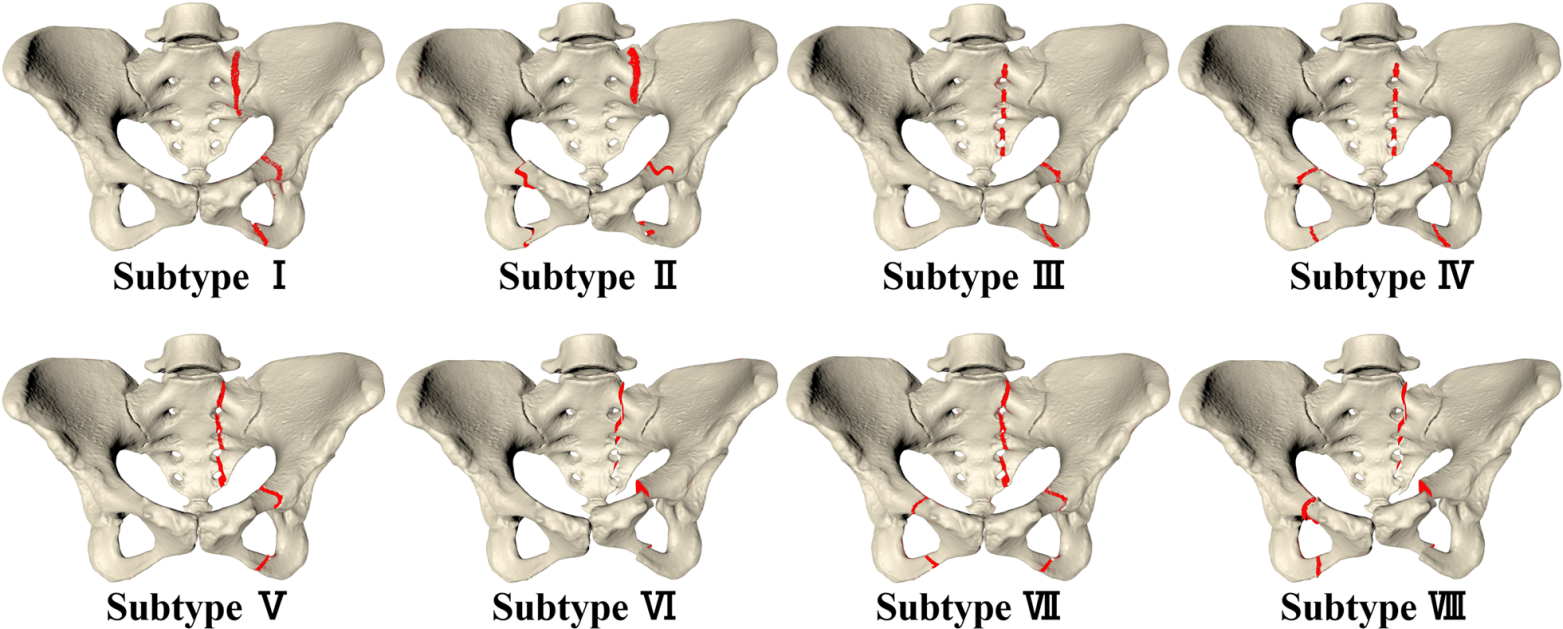
Illustrations of LC-1 pelvic fracture subtypes

According to the three main factors—the zone of sacral injury and completion, pubic ramus injury side, and the sagittal rotation of the injured hemipelvis— the LC-1 injuries were classified into the following eight subtypes, as shown in Fig. 2.

*Subtype I*: Compression fracture of sacrum zone 1 + fracture of the unilateral pubic ramus + without sagittal rotation deformity on the injured hemipelvis.

*Subtype II*: Compression fracture of sacrum zone 1 + fracture of the bilateral pubic ramus + without sagittal rotation deformity on the injured hemipelvis.

*Subtype III*: Incomplete fracture of sacrum zones 2/3 + fracture of unilateral pubic ramus + without sagittal rotation deformity on the injured hemipelvis.

*Subtype IV*: Incomplete fracture of sacrum zones 2/3 + fracture of the bilateral pubic ramus + without sagittal rotation deformity on the injured hemipelvis.

*Subtype *V: Complete fracture of sacrum zones 2/3 + fracture of the unilateral pubic ramus + without sagittal rotation deformity on the injured hemipelvis.

*Subtype VI*: Complete fracture of sacrum zones 2/3 + fracture of the unilateral pubic ramus + with sagittal rotation deformity on the injured hemipelvis.

*Subtype VII*: Complete fracture of sacrum zones 2/3 + fracture of the bilateral pubic ramus + without sagittal rotation deformity on the injured hemipelvis.

*Subtype VIII*: Complete fracture of sacrum zones 2/3 + fracture of the bilateral pubic ramus + with sagittal rotation deformity on the injured hemipelvis.

**Fig. 3:**
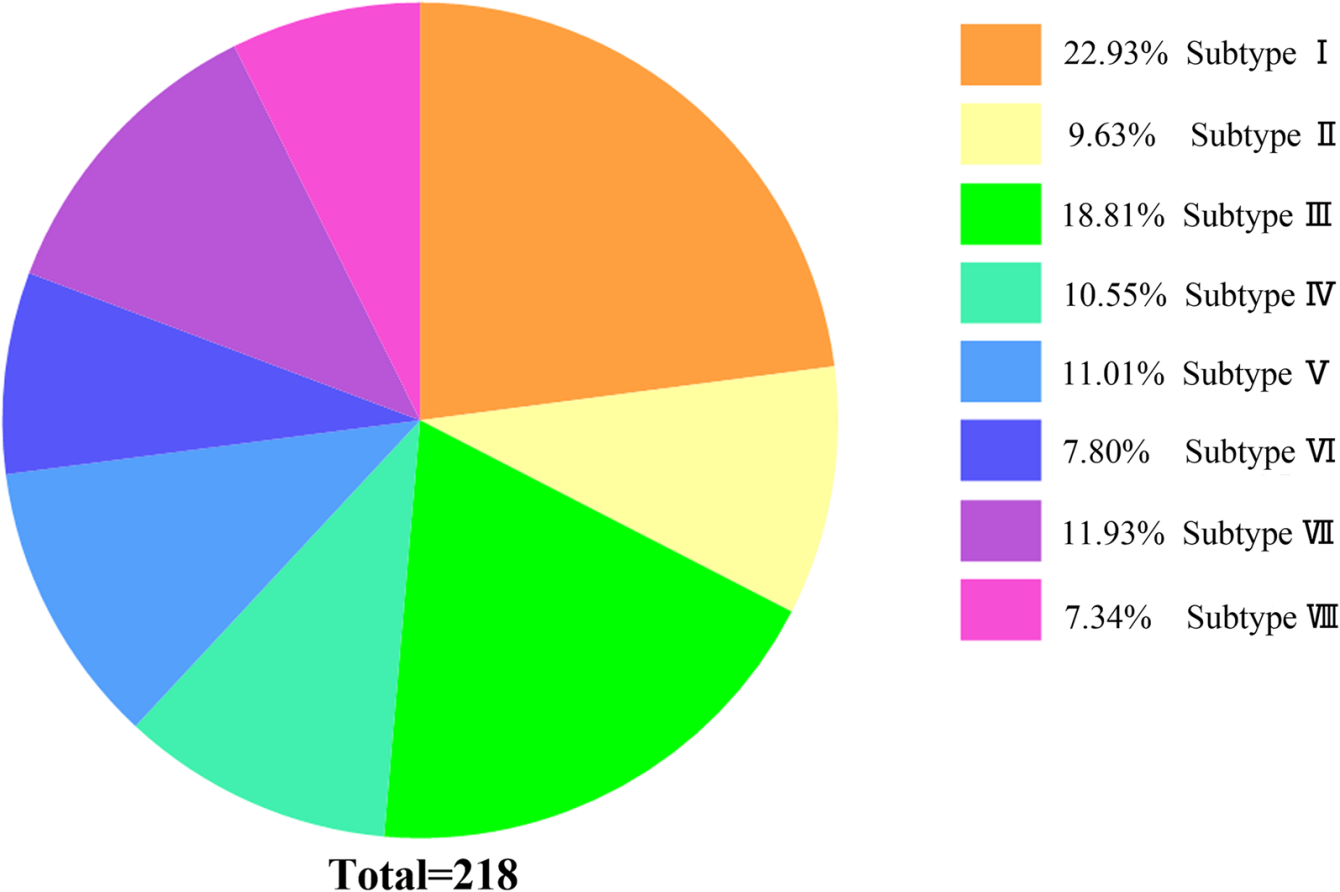
Proportions of eight LC-1 fracture subtypes

In particular, the rotated pelvis showed < 8° in sagittal plane rotation compared to that in the non-rotated hemipelvis. Illustrations of the subtypes are shown in Fig. 2, and their compositions are shown in Fig. 3.

### Finite element analysis based on pelvic ring stability

#### Von Mises stress distributions

**Fig. 4:**
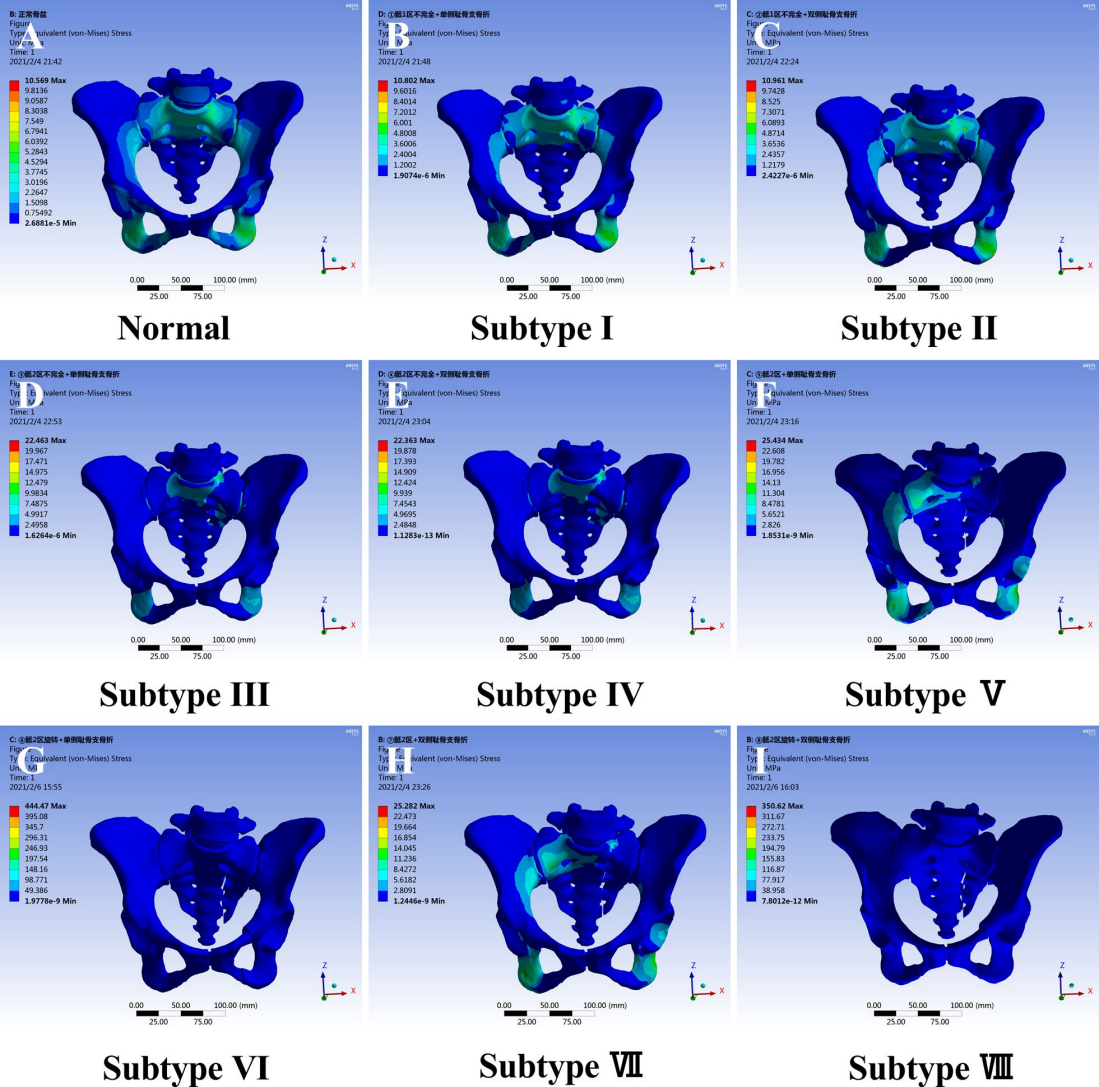
Von Mises stress distributions for the whole pelvis in LC-1 fracture subtypes (**A** normal pelvis; **B** subtype I; **C** subtype II; **D** subtype III; **E** subtype IV; **F** subtype V; **G** subtype VI; **H** subtype VII; **I** subtype VIII). The maximum stress was subtype VI and subtype VIII, but the illustrations were in Fig. 5

**Fig. 5:**
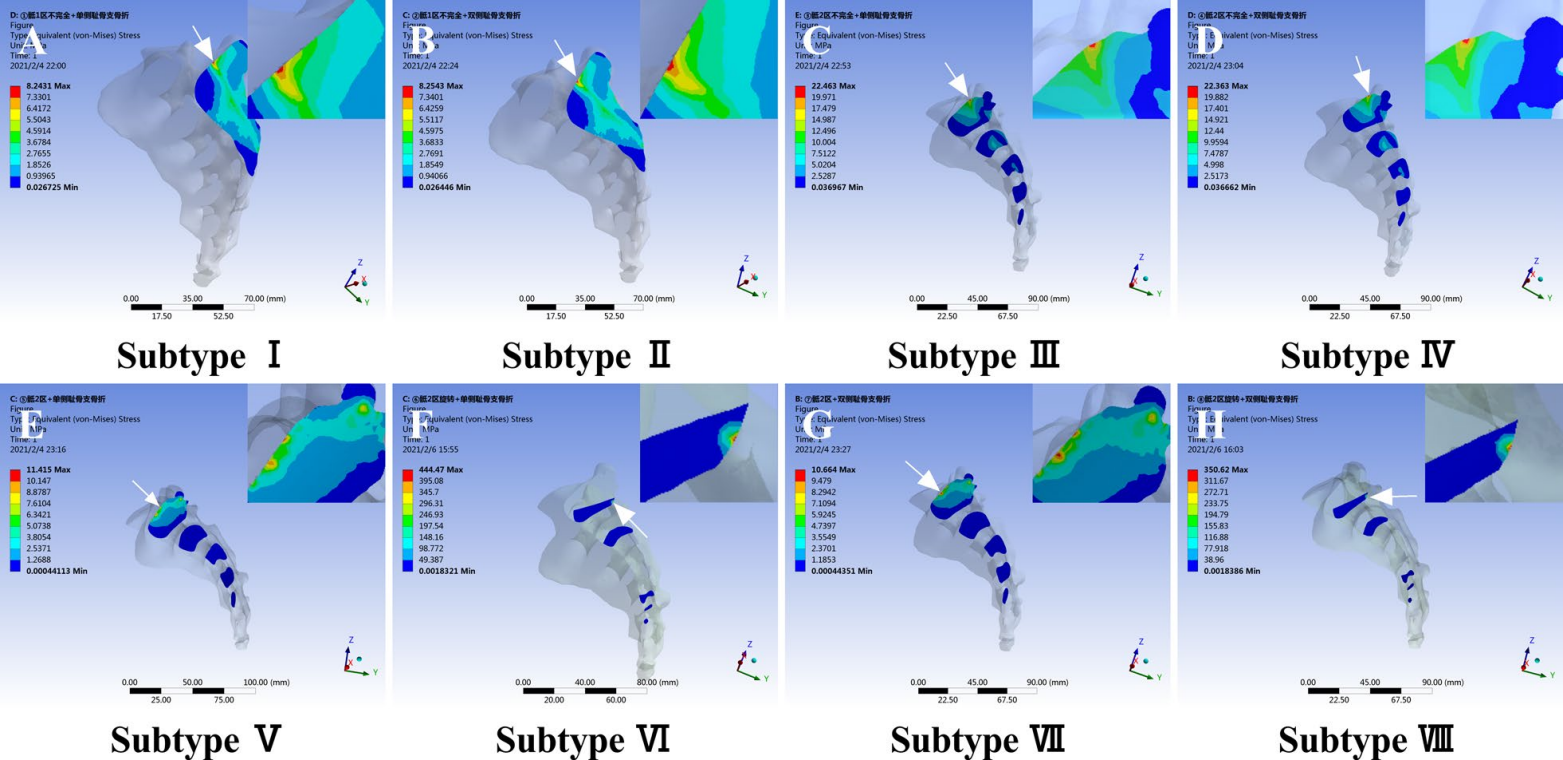
Von Mises stress distributions in the sacrum of LC-1 fracture subtypes (**A** subtype I; **B** subtype II; **C** subtype III; **D** subtype IV; **E** subtype V; **F** subtype VI; **G** subtype VII; **H** subtype VIII). The order of sacral stress was subtype VI/subtype VIII > subtype III/subtype IV > subtype VI/subtype VIII > subtype I/subtype II. The white arrow shows the site of maximum stress (red) in eight subtypes

The stress distribution diagrams of the normal pelvis and the eight fracture subtypes were shown in Fig. 4. In addition, the sagittal section of the sacrum along the fracture line is shown in Fig. 5.

The maximum stress of the normal pelvis was 10.57 Mpa. The maximum stress for subtypes I–VIII were 10.08 Mpa, 10.96 Mpa, 22.46 Mpa, 22.36 Mpa, 25.43 Mpa, 444.47 Mpa, 25.28 Mpa, and 350.62 Mpa, respectively. The order of pelvic stress was subtype VI/subtype VIII > subtype V/subtype VII > subtype III/subtype IV > subtype I/subtype II. Furthermore, there was no significant difference in stress between unilateral and bilateral public ramus fractures. The maximum stress was in the sacrum, showing Fig. 5.

Analysis of stress in the posterior pelvic ring showed that after compression fracture in sacral zone 1, compared to the normal pelvis, there was no significant difference in the stress distribution among normal, subtype I, and subtype II. The maximum stress of subtypes I and II (Fig. 5A, B) was approximately 8 Mpa and was located at the compression fracture of the sacrum, compared to 7 Mpa at this location in the normal pelvis. For incomplete fractures in sacral zones 2/3, the maximum stress in subtypes III and IV (Fig. 5C, D) increased to 22 Mpa, 3.6 times that in the normal pelvis. The peak also occurred at the site of the incomplete sacral fracture. For complete fractures in sacral zones 2/3, the total stress was 25 Mpa in subtypes V and VII (Fig. 4F, H), 4.2 times that in the normal pelvis. However, the sacral stress was approximately 10 Mpa at the fracture site, and the site of peak stress was in the ipsilateral ischial branch. For rotations occurring in sacral zones 2/3, which were categorized as subtypes VI and VIII (Fig. 5F, H), the maximum stress increased to 444 Mpa and 350 Mpa, respectively. The stress increases by approximately 60–70-fold compared to that in the normal pelvis. The order of sacral stress was subtype VI/subtype VIII > subtype III/subtype IV > subtype VI/subtype VIII > subtype I/subtype II, which differed from the order observed for total stress.

#### Total deformation

**Fig. 6:**
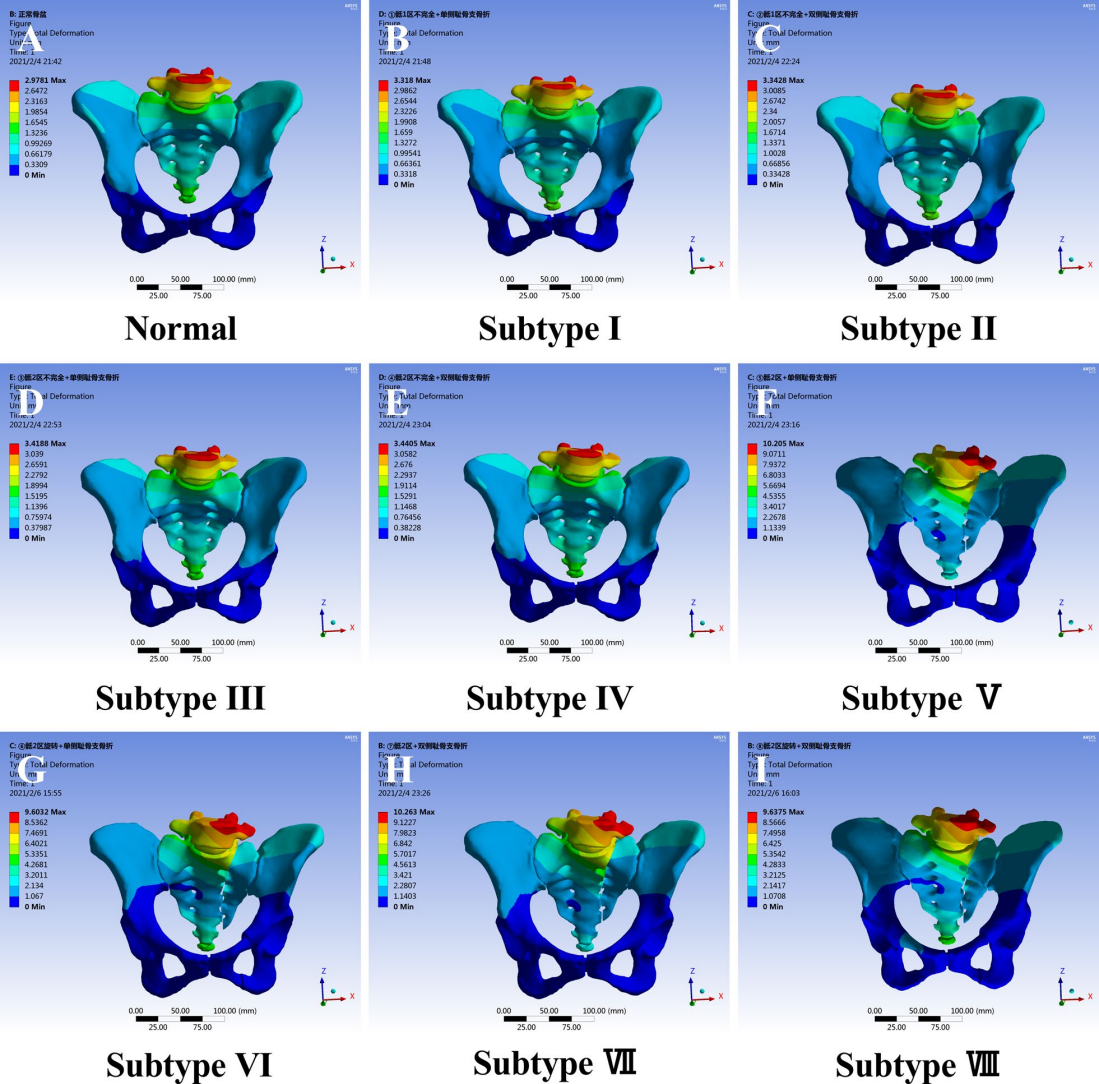
Total deformation distributions in the whole pelvis of LC-1 fracture subtypes (**A** normal pelvis; **B** subtype I; **C** subtype II; **D** subtype III; **E** subtype IV; **F** subtype V; **G** subtype VI; **H** subtype VII; **I** subtype VIII). The maximum total deformation was 10.2 mm for subtypes V (F) and VII (H)

The deformation diagrams of the normal pelvis and the eight fracture subtypes were shown in Fig. 6. The maximum deformation was observed at the L5 vertebra. Because of complete fractures in sacral zones 2/3, the deformation in subtypes V-VIII (Fig. 6F,–I) is unilaterally distributed, rather than symmetrically distributed in in subtypes I–IV (Fig. 6B–E). The maximum deformation of the normal pelvis was 2.9 mm (Fig. 6A). For subtypes I–IV (Fig. 6B–E), the maximum deformations were close to normal, at 3.3 mm, 3.3 mm, 3.4 mm, and 3.4 mm, respectively. However, the maximum deformation was 10.2 mm for subtypes V and VII (Fig. 6F, H), and 9.6 mm for subtypes VI and VIII (Fig. 6G, I). Thus, the order of total deformation was subtype V/subtype VII/subtype VI/subtype VIII > subtype III/subtype IV/subtype I/subtype II. There was no significant difference in total deformation between unilateral and bilateral public ramus fractures.

### Deformations in the sacrum and public ramus

The maximum deformation occurred at the L5 vertebra; thus, the deformations of the sacrum and public ramus were also determined.

**Fig. 7:**
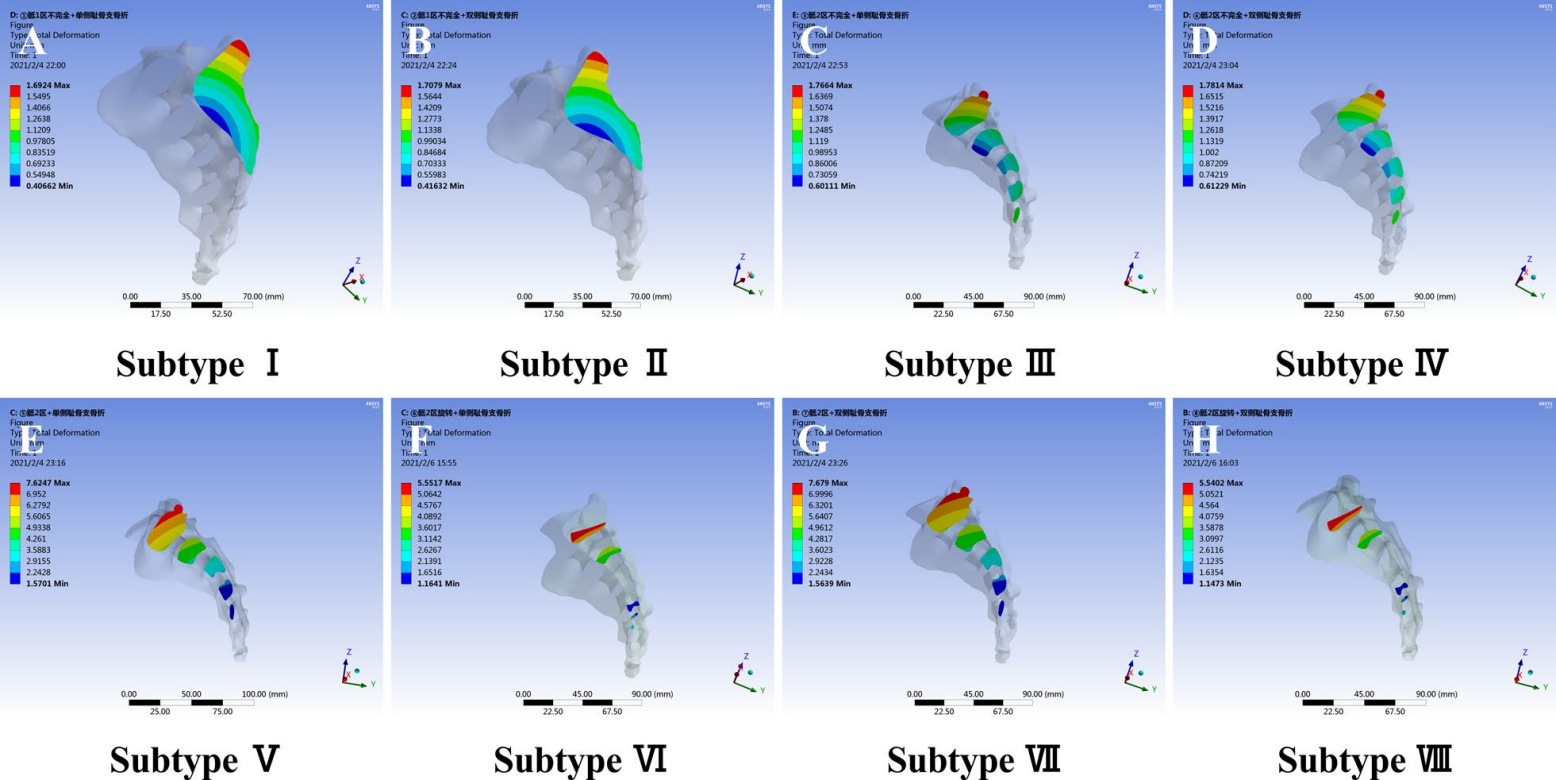
Total deformation distributions in the sacrum of LC-1 fracture subtypes (**A** subtype I; **B** subtype II; **C** subtype III; **D** subtype IV; **E** subtype V; **F** subtype VI; **G** subtype VII; **H** subtype VIII). The order of sacral deformation was subtype V/subtype VII/subtype VI/subtype VIII > subtype III/subtype IV/subtype I/subtype II. The maximum displacement of the sacrum may occur in subtypes V-VIII

The sagittal section of the sacrum along the fracture line is shown in Fig. 7. The maximum deformations were observed for subtypes V–VIII (Fig. 7E–H), at approximately 5.0–7.0 mm, while the deformations for subtypes I–IV were approximately 1.7 mm (Fig. 7A–D). The order of sacral deformation was subtype V/subtype VII/subtype VI/subtype VIII > subtype III/subtype IV/subtype I/subtype II. The results from the sacral deformation analysis suggested no significant difference between compression fracture of sacral zone 1 and incomplete fracture of sacral zone 2/3. After complete fracture of the sacral zone 2/3 or rotation hemipelvis, the deformation increased by 3–fourfold compared to that for subtypes I–IV. The maximum displacement of the sacrum maybe occur in subtypes V-VIII.

**Fig. 8:**
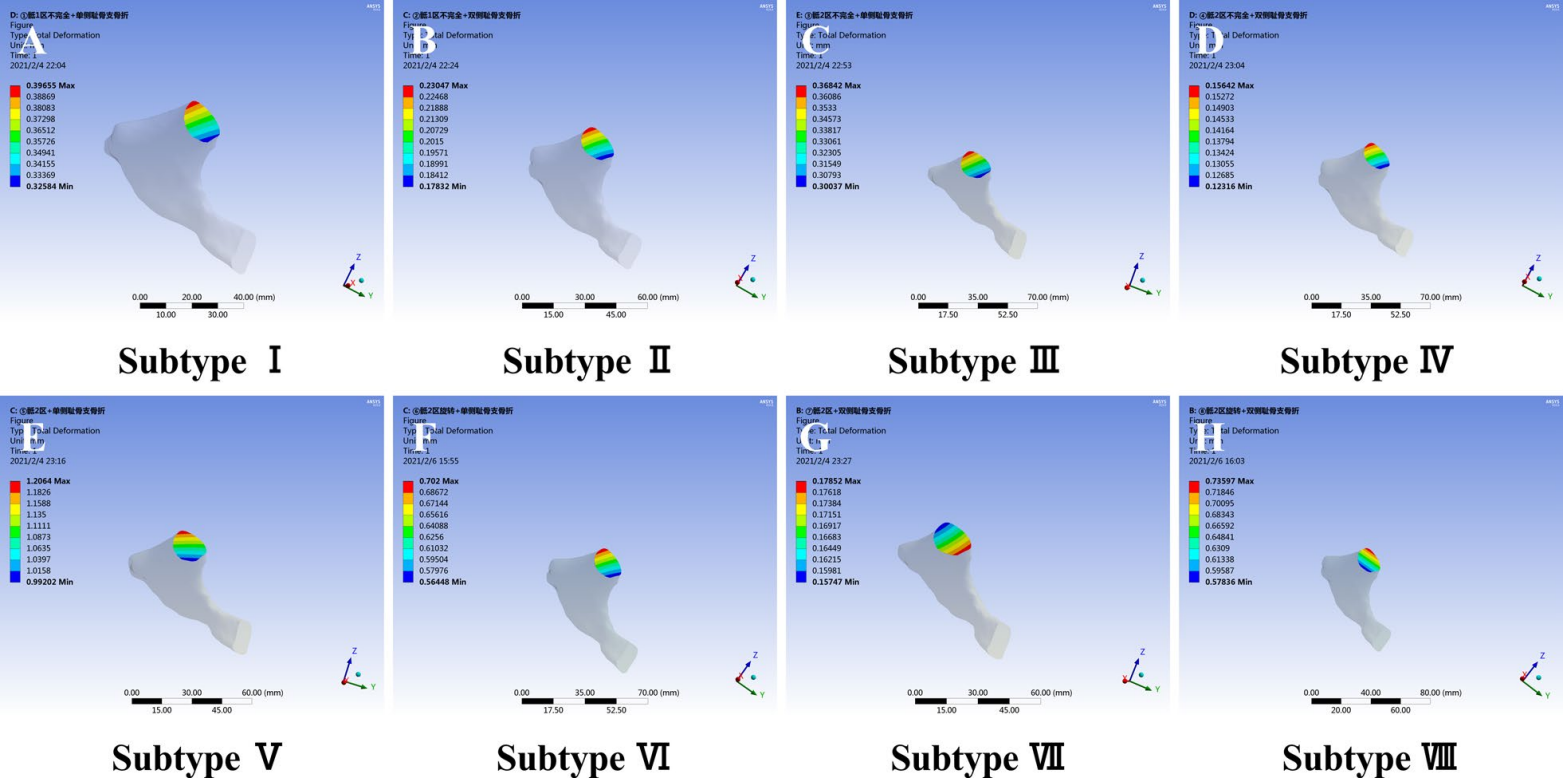
Total deformation distribution in the public ramus of LC-1 fracture subtypes (**A** subtype I; **B** subtype II; **C** subtype III; **D** subtype IV; **E** subtype V; **F** subtype VI; **G** subtype VII; **H** subtype VIII). There were no significant differences between different subtypes in deformation distribution in the public ramus. There was no obvious effect on the superior public ramus when LC-1 pelvic fracture

The sagittal section of the superior public ramus along the fracture line is shown in Fig. 8. There were no significant differences between subtypes. The deformation values ranged from 0.15 to 1.2 mm. The minimum and minimum deformations were observed for subtype IV and subtype V, respectively. There were no significant differences between different subtypes in deformation distribution in the public ramus. When loading 500 N, there was no obvious effect on the superior public ramus when LC-1 pelvic fracture.

## Discussion

LC-1 fractures are the most common pelvic ring injuries. However, they represent a heterogeneous spectrum of injury mechanisms and fracture patterns, resulting in a lack of solid evidence for a universal treatment algorithm [20]. This finite element analysis performed morphological mapping of the LC-1 fracture and classification of pelvic ring stability. We found that: (1) Unilateral public ramus fractures or bilateral injuries did not influence the stress distribution and deformation. (2) The maximum sacral stress occurred in subtypes VI and VIII. (3) Subtypes V–VIII showed the high possibility of whole-pelvis and sacral deformation. Thus, these results indicated that complete fracture of sacrum zone 2/3 might be a feature of unstable LC-1 fractures.

An increasing number of orthopedic surgeons have focused on exams under anesthesia to predict stability, a method that could increase agreement among experienced pelvic surgeons regarding the assessment of LC-1 pelvic ring stability and the need for definitive operative intervention [21]. Tosounidis et al. reported that LC-1 injuries with complete posterior sacral injury were inherently rotationally unstable and that patients presenting with these fracture patterns benefited from surgical stabilization [22]. This result is similar to that of the present study. However, even if the patients had a stable pelvic ring, the procedure under anesthesia could not be omitted before selecting an identified conservative treatment. In addition, there remains no clear consensus among surgeons regarding the method of performing exams under anesthesia or the definition of a positive exam under anesthesia [23]. The present finite element analysis provided a basic theory for stability in LC-1 fractures to increase confidence in distinguishing between stable and unstable fractures. In addition, regarding the relationship between sacral completeness and pelvic stability, a recent study reported that sacral fracture completeness in LC-1 pelvic ring injuries had weak interobserver reliability and significant potential for error by using sacral fracture completeness as a criterion to rule out occult instability [24]. That study examined lateral stress, in which two surgeons delivered maximal compressive force to the patient’s greater trochanters; thus, the force could not be quantitatively measured. Moreover, the LC-1 pelvic corresponded to the Tile-B2 type, which has been defined as rotationally unstable and vertically stable [25]. In addition, lateral stress was applied to the pelvis in their study. If the lateral pressure is sufficiently large, all LC-1 pelvic fractures will show instability. Thus, compared to lateral stress, vertical stress is more meaningful in assessing stability and can reflect stability when weight-bearing [16].

To our knowledge, this is the first study to divide LC-1 fractures into subtypes and perform finite element analysis to assess the stability of LC-1 fractures. We found that complete fracture of sacrum zones 2/3 may be a feature of unstable LC-1 fractures and identified the unstable subtypes for application in clinical practice. The maximum sacral stress was observed in subtypes VI and VIII, corresponding to the maximum total pelvic stress in the stress. In addition, the stresses for the whole pelvic and sacrum were consistent for subtypes III/IV and subtypes I/II. However, the peak stresses in the whole pelvis and sacrum were inconsistent in subtypes V and VII. In these models, the ipsilateral ischial branch was the site of peak stress. This is because the primary mechanical stress was decomposed when incomplete fractures developed to complete fracture in sacral zone 2. Subtypes V–VIII showed the maximum sacral deformation, which corresponded to the results of the results for total deformation.

Before performing the finite element analysis, we used patient CT data to morphologically map the fracture lines and classify fracture subtypes by machine learning. This process provided the basis for the accurate classification of LC-1 fractures. We observed stable subtypes I–IV and unstable subtypes V-VIII in approximately 61.92% and 38.08% of cases, respectively. The percentage of unstable LC-1 fractures was close to the 32.28% rate of patients receiving surgical treatment reported in a high-quality retrospective study [26], although this similarity may be a coincidence. Regardless, the system of subtypes could provide a reference for clinical practice. Furthermore, the eight subtypes were loaded with a vertical force of 500 N. The direction and magnitude of force are consistent with the weight-bearing status of the lower extremities of adults. Thus, our finite element analysis simulated this state to the greatest extent possible to provide a reference value.

Our study has some limitations. First, finite element analysis is a universal way of initially resolving the issue. It simulates the status, and its conclusions should be seen as a reference. Second, we input the identified Poisson's ratio ν and Young's modulus E of bones in this analysis and did not consider osteoporosis in the elderly. Thus, the results of this study are relevant for patients with normal bones. Third, the investigation mainly focused on bones and not the complex modeling of the ligaments. Moreover, all contacts between components were considered entirely bound to maintain pelvis integrity. Finally, further improvements are possible in the finite element analysis of LC-1 injuries.

## Conclusions

In conclusion, complete fracture of sacrum zones 2/3 may be a feature of an unstable LC-1 fracture. Surgeons should give surgical strategies for subtypes V–VIII in practice.

## Data Availability

The datasets generated and/or analyzed during the current study are not publicly available due to data privacy but are available from the corresponding author on reasonable request.

## Abbreviations

LC-1: Lateral compression
CT: Computed tomography
